# Cholera in Europe and India

**Published:** 1892-10

**Authors:** 


### Book Notices.

CHOLERA IN EUROPE AND INDIA, by Edward O. Shakespeare, A. M., M. D., Ph. D., United States Commissioner: Washington, Government Printing Office, 1890.

This magnificent volume is the report of the commission of one, constituted by Dr. Shakespeare, appointed by President Cleveland, in 1885, to go into the cholera districts of that year, with a view of personal study and investigation of the disease. It includes within its 900 or more pages a very considerable part of the literature of value upon the subject of cholera, issued up to the time of publication, besides the report of the commissioner upon the character of the surroundings of the disease foci visited, and the probable routes of its local and distant spread, as well as the results of the author's experimental work upon the subject, and what of therapeutic and preventive value he gleaned in his excursion.

In the first chapter a detailed account of the last wide-spread epidemic of cholera is given, occurring in Egypt, France, Italy, Spain, and in less degree in Great Britain, Germany, Austria, South America, Eastern Asia, and in a few cases in the port of New York. The second chapter is devoted to the topography and demography of British East India in relation to cholera, and is one of the most interesting in the volume, corroborating in the highest degree the general ideas as to the origin and spread of the disease in conditions of filth and overcrowding. Chapter III is occupied with bacteriological investigations upon, and the literature of, the ætiology and diagnosis of the disease, a considerable portion being taken up by the subject of cholera alkaloids or ptomaines. In the fourth chapter, Dr. Shakespeare details his personal observations upon the ætiology of the affection, and considers at some length the differentiation of cholera from malaria, acknowledging the hæmatozoon of malaria as a distinctive feature of the latter malady. The immunity of individuals is next considered, first, that granted by a previous attack of the affection, and second, that acquired through preventive inoculation, as prominently studied by Ferran, in Spain, several years since, and revived within the past several months, in a modified form by Brieger. A most important chapter details the author's views upon the matter of prevention of cholera, general and individual, in which the question of quarantine, particularly a national quarantine, is discussed. Dr.

Shakespeare's opinions upon this matter, as well as upon methods of individual prophylaxis, have received wide-spread attention during the past few weeks; and practically the same arguments and instructions as appear in the present volume, have been published at length by the author in the *Medical News*, of September 17th, of the current year.

As a conclusion, an admirable, general article upon cholera is, with propriety, presented, dealing with the nature, ætiology and pathology, symptomatology, diagnosis, prognosis and treatment of the affection. It is unfortunate that a more extended and more fully descriptive review of the work is precluded by want of space. The times are ripe for a thorough examination of so complete an exposition of the subject in hand, and the excellence of the production of Dr. Shakespeare makes the closest investigation of his labors pleasurable to his readers. The wisdom of the Presidential provision, enabling the prosecution and completion of such undertakings, is eminent, and stands forth as an act of executive philantrophy, the brighter, because of the studied tendency on the part of the government to disregard national human health, in comparison to a studied regard for the health of the beasts of the field within the limits of the republic.

As in most government publications, the typographical work is fairly, but not excellently, done; the illustrations are well executed, although the choice of subjects for illustration is not always apparently a happy one. A. J. S.

